# Supplementary material for: Dosage-Dependent Gynoecium Development and Gene Expression in Brassica napus-Orychophragmus violaceus Addition Lines
Source: Plants (Basel). 2021 Aug 25;10(9):1766. doi: 10.3390/plants10091766 (PMC8469106; doi:10.3390/plants10091766)
Supplement: Supplementary file 1 [file plants-10-01766-s001.zip › Supplementary figures.pdf]

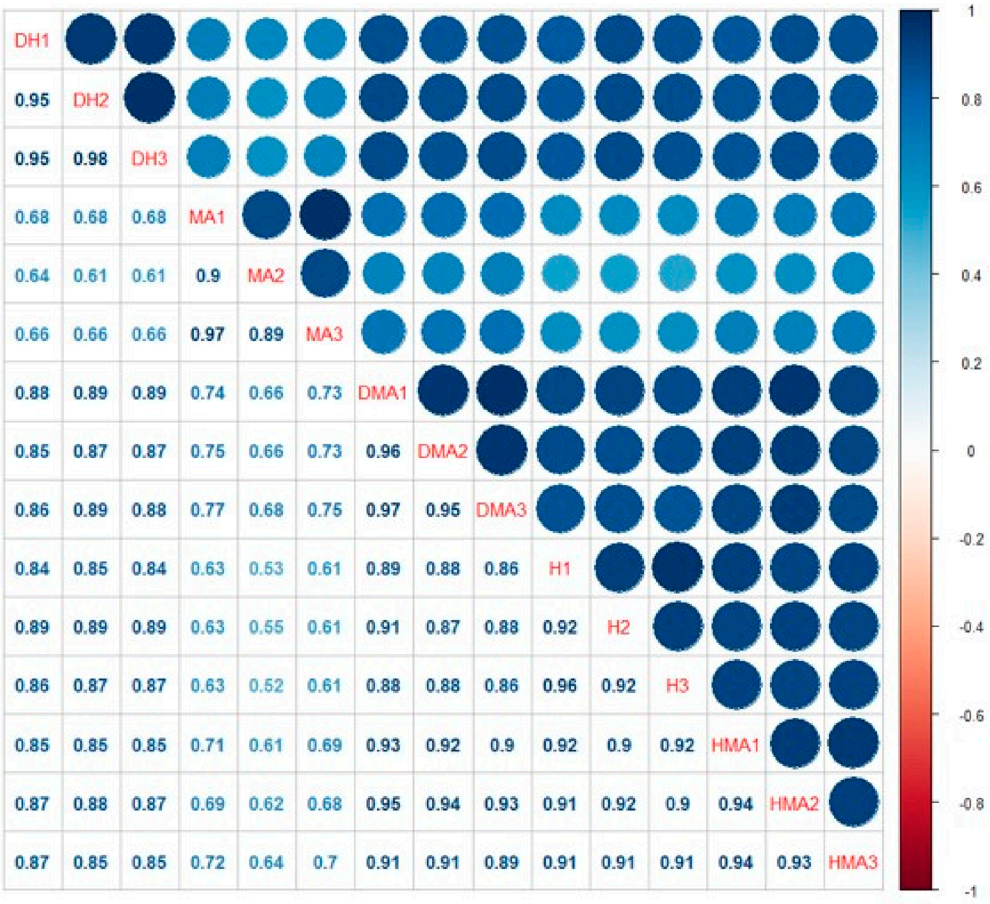

Figure S1 Pearson correlation coefficients between each pair of biological replicates under same sampling conditions.

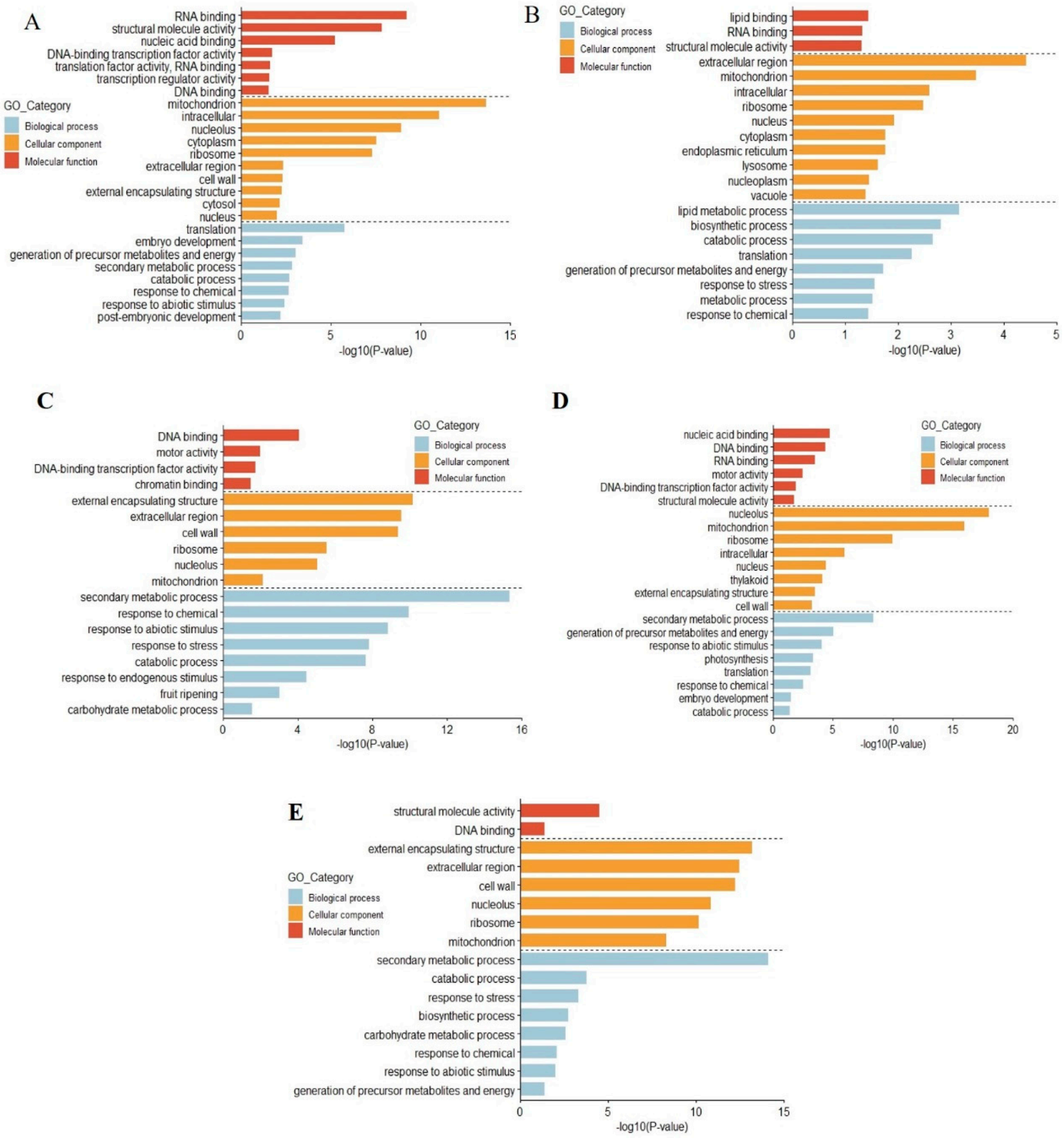

Figure S2 GO enrichment analysis. A-E, GO enrichment analysis on DEGs between DA-vs-DH; HMA-vs-H; MA-vs-DA; MA-vs-DH; MA-vs-HMA, respectively. DEGs are grouped to the secondary classification of hierarchical GO terms.
