# Supplementary material for: Dosage-Dependent Gynoecium Development and Gene Expression in Brassica napus-Orychophragmus violaceus Addition Lines
Source: Plants (Basel). 2021 Aug 25;10(9):1766. doi: 10.3390/plants10091766 (PMC8469106; doi:10.3390/plants10091766)
Supplement: Supplementary file 1 [file plants-10-01766-s001.zip › Supplementary Tables.pdf]

Table S1 Statistics of RNA-seq reads and mapped reads

Samples[a]	Total raw reads	Total clean reads	Total clean nucleotides	Mapped reads	Uniquely mapped
**DH-1**	15,106,700	14,349,164	2,093,553,321	87.33%	76.67%
**DH-2**	19,586,084	18,730,890	2,726,917,544	87.46%	76.95%
**DH-3**	16,054,762	15,243,337	2,223,356,614	87.38%	76.90%
**MA-1**	18,848,316	17,924,483	2,607,613,394	86.95%	76.15%
**MA-2**	20,286,769	19,114,694	2,791,775,684	86.61%	75.43%
**MA-3**	16,584,353	15,893,453	2,308,187,234	87.14%	76.33%
**DA-1**	27,377,463	26,066,891	3,861,544,027	85.59%	65.48%
**DA-2**	27,031,150	25,841,520	3,827,558,168	85.70%	65.47%
**DA-3**	26,404,691	25,036,819	3,708,816,187	86.47%	67.78%
**HA1**	30,953,071	29,563,188	4,388,826,269	86.66%	67.28%
**HA2**	29,399,076	28,365,551	4,220,043,973	87.40%	67.14%
**HA3**	29,741,537	28,690,902	4,268,684,704	87.52%	67.42%
**HMA1**	30,550,165	29,453,168	4,378,847,876	86.69%	66.12%
**HMA2**	29,697,642	28,526,829	4,243,329,841	85.91%	64.61%
**HMA3**	32,657,079	31,462,323	4,679,604,213	86.63%	66.23%
**Total**	370,278,858	354,263,212	52,328,659,049	-	-
**Average**	24,685,257.2	23,617,547.47	3,488,577,270	86.76%	70.40%

[a]1, 2 and 3 are three biological replicates; DH represent AACC, MA represent AACC+ 1 I_O, DA represent AACC+ 2 I_O, HA represent AC, HMA represent AC+ 1 I_O.

Table S2 DEGs for brassinosteroid biosynthesis in each comparison

Gene name	*Arabidopsis* gene ID	*B. napus* gene ID	Differentially expressed (Y) or not (N) in each comparison				
			HMA vs HA	MA vs DH	DA vs DH	MA Vs DA	MA vs HMA
CAS1	AT2G07050	BnaA03G0388800ZS	N	Y ↓	N	Y ↓	Y ↓
		BnaC03G0481600ZS	N	Y ↓	N	N	N
SMO1-1	AT4G12110	BnaA02G0263800ZS	N	Y ↑	N	Y ↑	Y ↑
		BnaC02G0358100ZS	N	Y ↑	N	Y ↑	Y ↑
FACKEL	AT3G52940	BnaA07G0182200ZS	N	N	N	N	Y ↓
SMO2-1	AT1G07420	BnaC05G0053700ZS	N	N	N	N	Y ↓
		BnaA08G0304100ZS	N	N	N	N	Y ↓
		BnaC08G0028800ZS	N	N	N	N	Y ↓
DWF5	AT1G50430	BnaC03G0782500ZS	N	Y ↓	N	Y ↓	Y ↓
DWF1	AT3G19820	BnaC01G0393200ZS	N	N	N	Y ↓	Y ↓
		BnaC05G0394400ZS	N	Y ↓	N	Y ↓	N

Note: ↓ and ↑ indicated that the gene was dow- and up regulated, respectively.
